# Tumour of the Rectum, Containing Portions of a Fœtus, Extracted from a Child Six Years Old

**Published:** 1851-04

**Authors:** 

**Affiliations:** Head Surgeon to the Hospital of La Charité, Lyons


*Tumour of the Rectum, containing Portions of a Fœtus, extracted from a Child six years old.* By M. Bouchacourt, Head Surgeon to the Hospital of La Charité, Lyons.—Catharine Sery, a girl 5½ years of age, after having suffered from several disorders connected with the digestive organs, passed a large quantity of purulent matter from the anus. The discharge continued for seven months: the child fell away, and the excretion of fæces became more and more difficult.

About a fortnight after the first discharge of purulent matter, that is near the end of August, 1849, a long lock of auburn-colored hair was passed from the anus; it bore great resemblance to that of the little patient. About the end of March, 1850, the purulent discharge ceased, and nothing unusual was noticed until the 17th April, when some blood was passed from the anus; the child now suffered from constant tenesmus. As the efforts increased a tumor presented itself at the orifice of the anus, disappearing again when the strain ceased. The patient was now admitted into hospital, under the care of M. Bouchacourt.

On examining the rectum it was found that the tumor extended some way into the gut, adhering to the posterior wall only at its upper part. The tumor was soft at some points, hard at others, smooth and partly covered with hair. Its nature thus became manifest at once; it was resolved that we should extirpate it, and nature assisted us, for after a long walk the tumor protruded almost entirely from the rectum. A careful examination now showed that the root or pedicle of the tumor did not contain any vessels, and that no portion of the intestinal canal was connected with it. Extirpation was therefore easily effected by means of a double ligature and the scissors. A few drops of blood only escaped, and the child was well in a few days.

The tumor was an irregularly oval mass, weighing 2½ oz., covered with fine white skin, exactly like that of a new born child. On one end were some hairs, and on the other a fine down. On making a very careful examination, a transverse fissure was discovered close to one of the ends, and on a tubercle attached to the lower lip of the fissure were two teeth, a canine and a molar one. On a similar tubercle of the upper lip was an incisor tooth, placed transversely. On dividing the body of the tumor, the first object which presented itself was a round bone, imbedded in fibro-fatty matter. This bone supported a number of teeth and some capsules. Beneath this bone was another small one of irregular shape, the nature of which it was impossible to determine. It might be a rudimental rib or vertebra. Finally the lower portion of the trunk was represented by a cartilaginous mass of the size and shape of a small nut.—*London Med. Times, 1st March,* 1851, *from Bulletin de l'Institut.*

